# HAND GESTURES AND HOW THEY HELP CHILDREN LEARN

**DOI:** 10.3389/frym.2018.00029

**Published:** 2018-06-26

**Authors:** Sharice Clough, Caitlin Hilverman

**Affiliations:** 1 University of Iowa, Iowa City, IA, United States; 2 Vanderbilt University Medical Center, Nashville, TN, United States

When we talk, we often make hand movements called gestures at the same time. Although just about everyone gestures when they talk, we usually do not even notice the gestures. Our hand gestures play an important role in helping us learn and remember! When we see other people gesturing when they talk—or when we gesture when we talk ourselves—we are more likely to remember the information being talked about than if gestures were not involved. Our hand gestures can even indicate when we are ready to learn new things! In this article, we explain how gestures can help learning. To investigate this, we studied children learning a new mathematical concept called equivalence. We hope that this article will help you notice when you, your friends and family, and your teachers are gesturing, and that it will help you understand how those gestures can help people learn.

## WHAT KINDS OF HAND GESTURES ARE THERE?

We all make spontaneous hand movements, called gestures, when we talk. These **co-speech gestures** are produced in rhythm with our speech and are related to the meaning of what we are saying. For example, when talking about attending a piano recital, you might move your fingers right and left in front of you to illustrate what playing a piano looks like.

There are several different types of gestures that serve different purposes. Some gestures describe objects or actions, like the piano example above. These are called **iconic gestures**, because they create a picture. Sometimes our gestures do not make pictures, but they move in rhythm with our speech. These are called **beat gestures**, because they follow the beat of our speech. For example, you might flick both wrists downward when saying the word amazing in the following sentence: “The recital was amazing.” These gestures can help the speaker emphasize certain words. **Deictic (DIKE-tick) gestures** help the speaker direct the listener’s attention by pointing something out. For example, at a recital, you might use your forefinger to point toward the pianist when whispering to your friend about a song that you particularly enjoyed.

These terms for the different types of gestures are useful for the people who study gestures, including psychologists, cognitive scientists, linguists, and neuroscientists. These scientists often study related ideas: psychologists study thinking and human behavior, cognitive scientists study thinking and learning, linguists study language and communication, and neuroscientists study the structure and function of the brain. Many different researchers are beginning to study co-speech gestures, because they can tell us more about what is happening in people’s minds.

**FIGURE 1:**
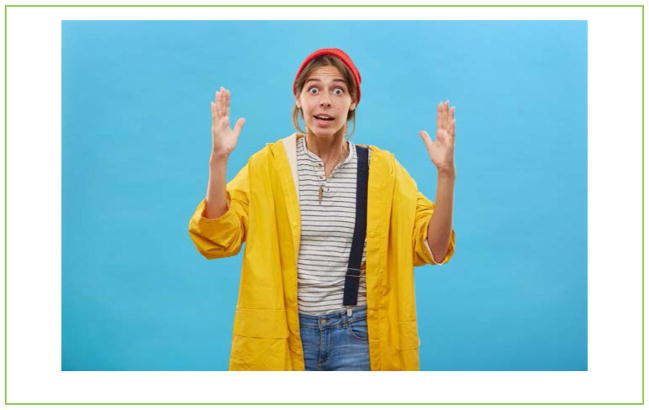
By gesturing about the fish that she caught last week, this woman shows us exactly how big the fish was.

Even though gestures are related to speech, they can sometimes show us information that is not present in speech. For example, if I said, “I caught a fish last weekend,” you would not know how big it was. What if, while telling you I caught a fish, I put my hands out 18 inches apart? (Figure 1). You would know I caught a big fish, even though I did not say anything about its size! People use gestures to convey information that is not present in speech, and they sometimes do this without even realizing it. The listener uses the unstated information in the gesture to understand what is being said. Gestures can be helpful to both the listener viewing the gestures and to the speaker producing them, and we are going to tell you how.

## HOW DO GESTURES HELP THE LISTENER?

Because gestures can show information that is not present in speech, researchers have tested whether gestures help us understand what a speaker is saying. This has been tested by many different researchers using a simple method. To test the importance of gestures to understanding, researchers compare two groups of people—one group that views someone talking *and* gesturing and a second group that views someone talking *without* gesturing. Then they test whether the people in the group that sees gestures learn better than those in the group that do not see gestures. What the research usually finds is that seeing gestures while hearing someone talk helps the listener remember that information better. One researcher performed a **meta-analysis** of this work [1]. In a meta-analysis, researchers combine the results of many studies investigating similar questions. This allows researchers to make conclusions with more confidence, because they are looking at a lot of data from different studies. So, this researcher compared the findings of 38 of these studies, to try to understand when gestures can be most helpful for learning. She found that gestures helped people learn better when the gestures represented motor (movement) actions (such as throwing a baseball) than when gestures were abstract (such as clenching a fist to represent *anger*). Motor gestures are iconic, because they demonstrate how to do something. When children and adults saw iconic gestures with speech, they understood the message better and remembered it more than did people who only heard speech but did not see gestures. And although gestures helped everyone learn and understand, they were extra helpful for children!

By testing math learning, researchers have studied how children learn through gestures. One such study investigated whether second, third, and fourth graders learned math better when their teacher gestured [2]. The teacher taught the students how to solve a math problem about **equivalence**. An example of an equivalence problem is shown here:

4+3+6=_+6

Students had to decide what number to put in the blank, to make the right side equal to the left. For example, in the above equation, the number seven belongs in the blank, so that both sides equal 13. The teacher taught half the kids using just speech. In this condition, the teacher verbally explained that in an equivalence equation, “*one side is equal to the other side*.” The teacher taught the other half of the kids using speech *and* gestures. Whenever she said, “one side,” she pointed to the left side of the equation with a sweeping gesture. Whenever she said, “the other side,” she pointed to the right side of the equation with a sweeping gesture.

All students were then tested on the equivalence problems twice: Posttest 1 was immediately after learning and Posttest 2 was 24 h later. The students were also given a Transfer Test. A transfer test is designed to test students’ ability to apply their knowledge in new ways. The researchers wanted to determine whether the children could extend their knowledge of equivalence to other types of problems using similar rules. For example, third and fourth graders solved a multiplication equivalence problem such as:

3×4×2=_×4

Second graders, who were not quite ready for multiplication, solved more complicated addition problems on the Transfer Test, which did not have any of the same numbers on both sides, such as:

7+2+5=9+_.

**FIGURE 2:**
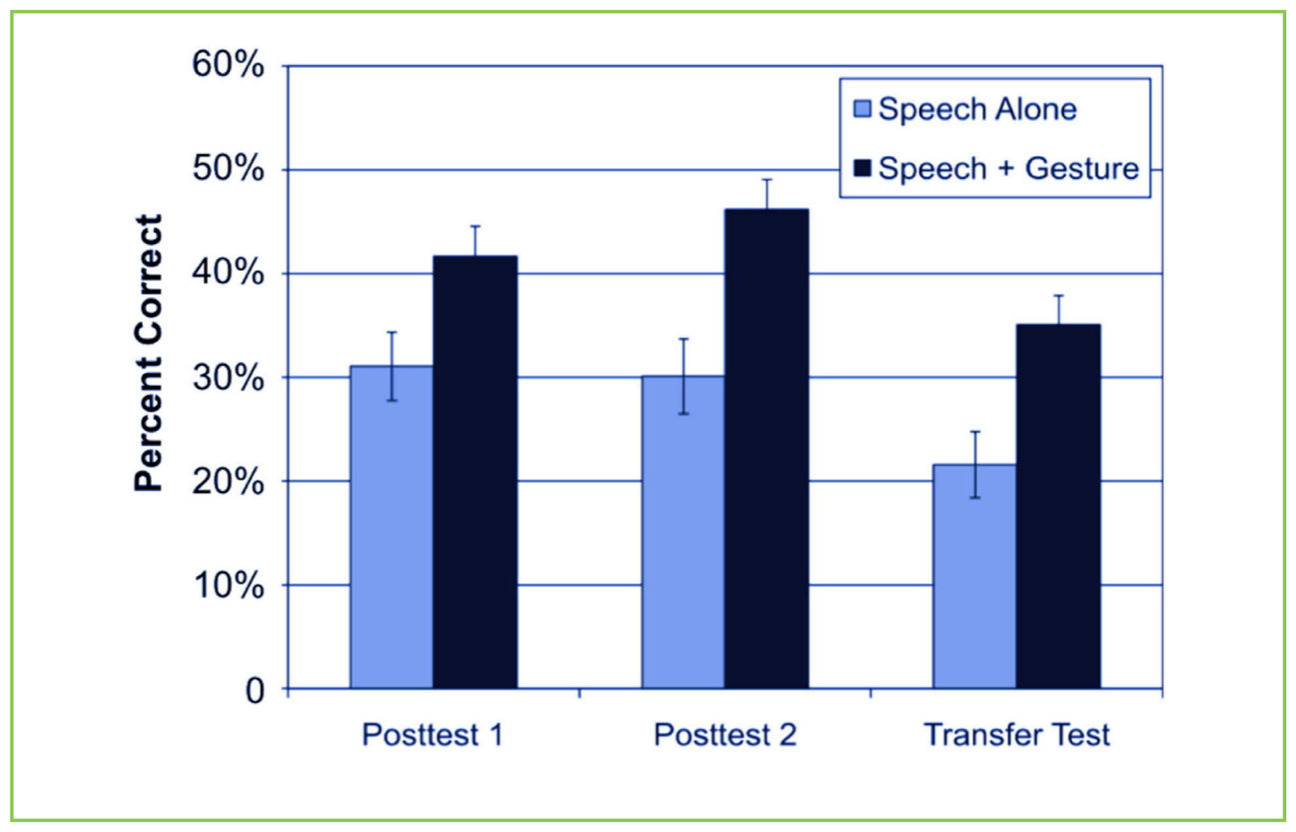
When teachers taught equivalence problems using both speech and gestures, children did better when tested on similar problems later. Children who were taught with gestures did better than children who learned without gestures immediately after learning (Posttest 1), 24 h later (Posttest 2), and on a Transfer Test that tested their ability to use the same rules to solve new problems (for example, using multiplication problems instead of addition). The height of each bar represents the average test scores for students in both conditions. The vertical lines at the top of each bar are error bars that represent how spread out scores are around the average. Larger error bars mean that scores are more spread out. Smaller error bars mean that scores are less spread out and closer to the average. Scientists have more confidence in their findings when their error bars are smaller. This figure is adapted with permission from Cook et al. [2].

The scientists compared the number of correct answers from children who saw gestures to those of the children who were taught with only speech. On Posttest 1, Posttest 2, and the Transfer Test, the students who saw gestures scored better than the students who only learned with speech. Figure 2 shows the difference between these two groups of children. Furthermore, students who learned with gestures also performed significantly better on Posttest 2 than they had on Posttest 1. This showed that gestures not only helped students learn better but gestures also helped their performance get better over time. Students who learned with only speech did not perform better on Posttest 2 than they did on Posttest 1.

## HOW DO GESTURES HELP THE SPEAKER?

Not only do students learn better when their teacher gestures but they also learn better when they use gestures themselves. A group of scientists studied whether third and fourth graders learned math better when they gestured during class [3]. The teachers taught the students how to solve addition equivalence math problems like the ones above. One group of students repeated the teacher’s words, “*I want you to make one side equal to the other side*.” A second group repeated the teachers’ gestures but *not* her words: they used one hand to point first to the numbers on the left side of the equation and then used the other hand to point to the numbers on the right side of the equation. A third group repeated the teacher’s gestures *and* words. After instruction, all students took a posttest that consisted of new addition equivalence problems. Then, 4 weeks later, all students completed a follow-up test with similar types of questions. The follow-up test allowed the researchers to test if gestures helped learning over time.

**FIGURE 3:**
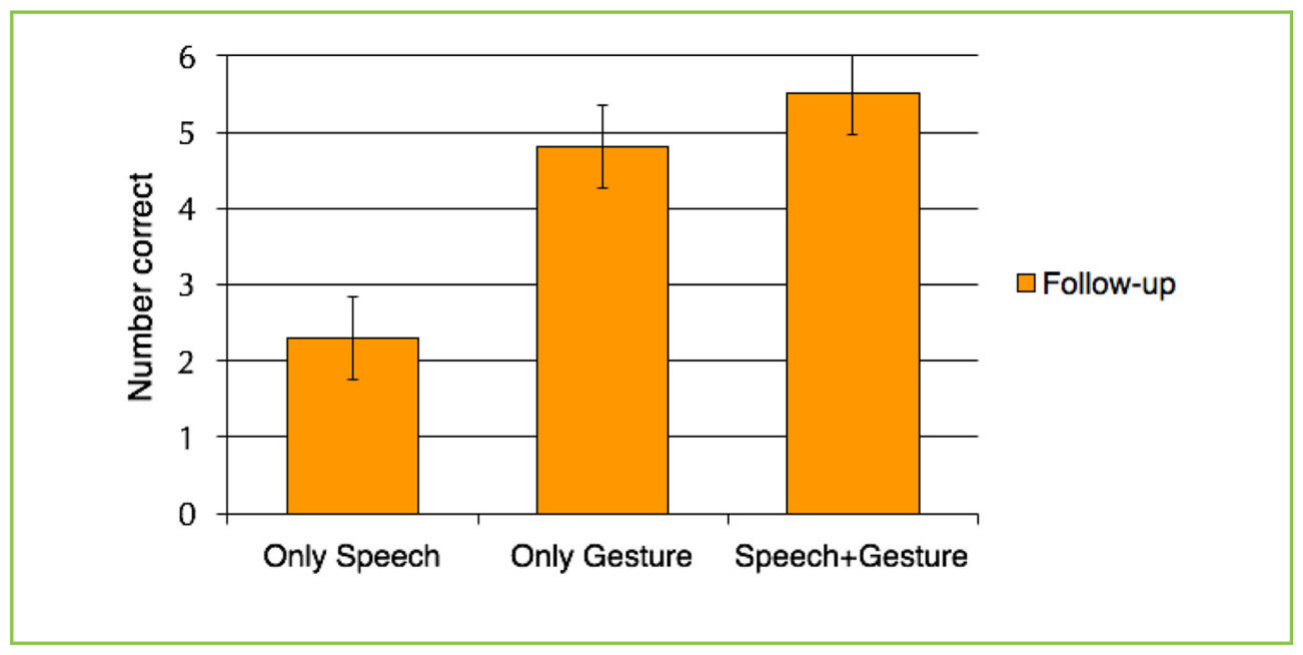
Each bar represents the average number of correct answers on the follow-up test for one of the three conditions. Children who learned with gestures or with speech and gestures combined performed better on the follow-up test 4 weeks later than children who learned with just speech.

Students from all three conditions performed similarly on the posttest that they completed right after learning: making gestures when learning did not benefit learning immediately. However, on the follow-up test 4 weeks later, the students who learned by only making gestures and the students who learned by making gestures along with speech performed better than the students who learned only by speech (Figure 3). Gesturing helped the students’ learning last over time, while the students who learned only with speech did not get this benefit. The researchers think that gesturing during learning helped the children produce stronger memories, because while gesturing they activated motor areas of the brain that were not activated in children who did not gesture.

## CAN GESTURES SHOW US WHEN CHILDREN ARE READY TO LEARN?

We have now shown you that gestures can improve learning, both when children see other people using gestures and when the children themselves use gestures. Is there something about gestures that can help us know when children are *ready* to learn something new? This seems to be the case for babies learning language. Before babies can talk, they communicate by pointing. Between 9 and 12 months, babies begin pointing to request objects like a bottle or a toy. The use of these early gestures tends to predict the first words that babies say a few months later. Babies usually say their first word at about 12 months. Before babies begin to combine multiple words together, they combine words and gestures. For example, a baby might point to a ball and say “mine” to indicate that the ball belongs to them. These combinations are called *gesture-speech mismatches* because the information in speech and gesture is different, or mismatched. By contrast, an example of a gesture-speech match would be the baby pointing to the ball and saying “ball.” Gesture-speech mismatches are a good thing because they show the baby is combining two ideas. In fact, these mismatches predict how well the babies will learn language: babies who produce more of these gesture-speech mismatches when they are 18 months old go on to produce more complex sentences when they are 3 years old! [4].

In these cases, gestures tell us what the child already knows and help us predict when the child will learn new words. Researchers were curious about whether gestures predicted learning in school-age children as well. The researchers studied the gestures children made when explaining how they solved mathematical *equivalence* problems, like the ones described earlier [5]. The researchers thought that, even though children might not be able to communicate how to solve these problems using speech, they might express some knowledge of equivalence in their gestures. For example, for the problem 4 + 3 + 6 = __ + 6, an 8-year-old child might *say*, “I added all the numbers and got 19.” This shows that the student does not understand the concept of equivalence, because the equal sign symbolizes that there are two separate sides that needed to be made equal. However, it might be evident from their *gestures* that the children are beginning to understand that the two sides of the equation are separate units. For example, the student might sweep his or her hand first under the left side of the equation and then under the right side, like the teachers did in the experiment above. The researchers wondered whether gesturing toward each side of the equal sign separately was signaling that the child was beginning to understand the concept of equivalence but could not yet form the idea into words.

To test this, the researchers gave third and fourth graders a test of mathematical equivalence problems before they had learned how to solve them. When they were solving these problems, the students were asked to explain how they got their answers. Then, the students who did not get any questions correct on the pretest were taught how to solve equivalence problems. The teacher did not gesture but guided the students through the problems and asked them to again explain how they got their answers. Students then took a posttest to assess how well they had learned.

**FIGURE 4:**
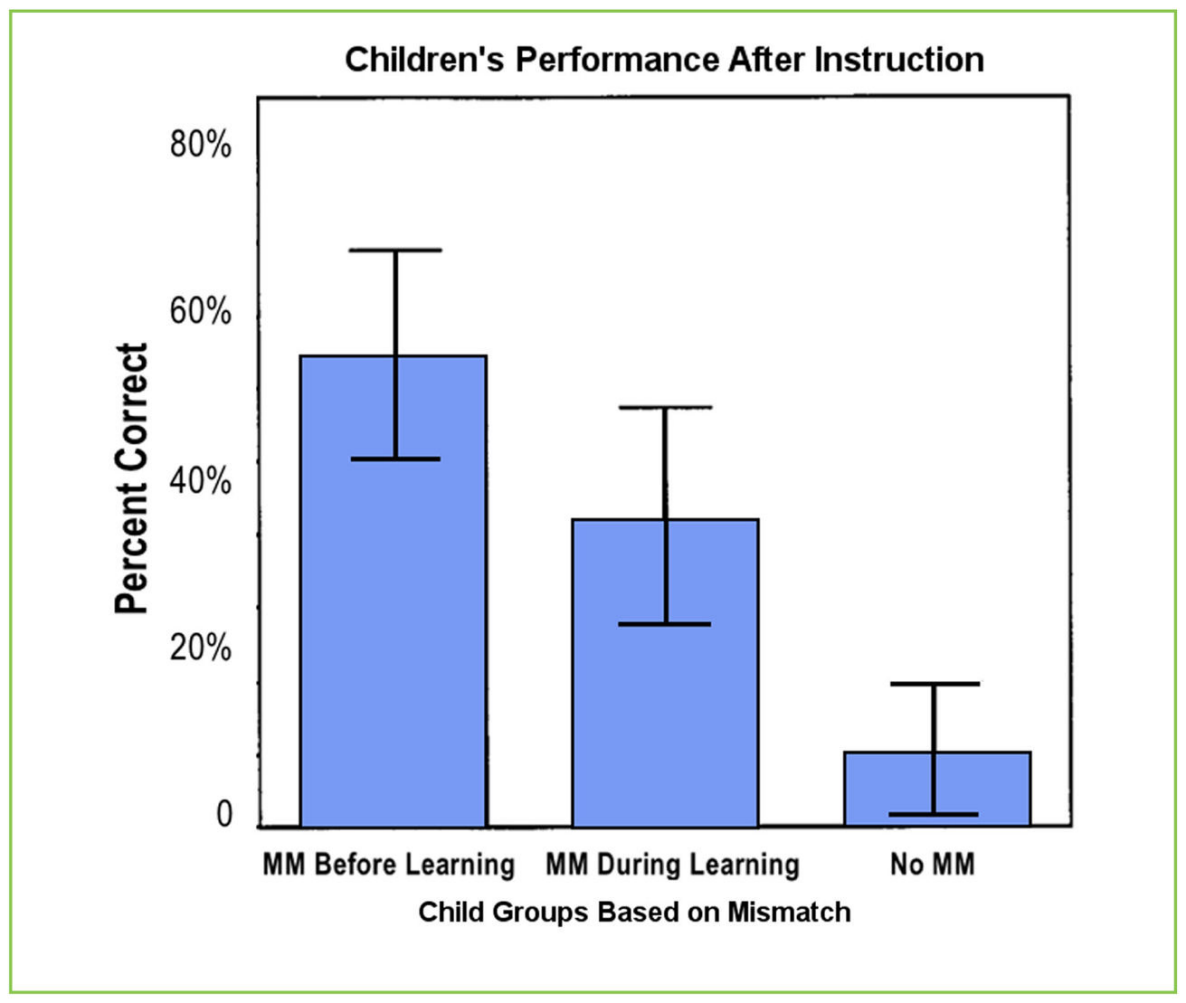
Children who produced gesture-speech MMs learned better than those who did not. Children who produced MMs before and during learning had higher average scores on the posttest than did students who produced no MMs at all. Abbreviation: MM, mismatch. This figure is adapted with permission from Goldin-Meadow and Singer [5].

The researchers then examined the students’ explanations for gesture-speech mismatches, like the hand-sweeping motion described above. Some children did use gesture-speech mismatches before and during learning. Those students provided two solutions: one in speech and another in gestures. The children were more likely to express the correct solution in gestures than they were in speech. The researchers then wanted to see if the children who used gesture-speech mismatches performed better on the posttest than did children who did not use gesture-speech mismatches during learning. The researchers compared the performance of three groups: (1) students who used gesture-speech mismatches when explaining their solutions before learning; (2) students who started to produce mismatches later, during learning; and (3) students who did not use any gesture-speech mismatches at all. Even though all the children started out providing incorrect solutions in speech, the children who used gesture-speech mismatches before learning and during learning got more answers correct on the post-test than did the children who did not use any gesture-speech mismatches (Figure 4).

Similar to the way babies communicate different information in their gestures and in their speech, children can do the same when explaining math problems. The researchers thought that mismatches between gestures and speech demonstrated that the children were beginning to understand the concept of equivalence but could not yet put it into words. All the children needed was a little instruction from a teacher and they successfully completed equivalence problems on the posttest. Children who did *not* show these gesture-speech mismatches were not really helped by the teacher’s instruction, and they got very few problems correct on the posttest. Not only can gestures help children learn but also children’s spontaneous gestures can show us when they are beginning to grasp a new concept!

## EXPLORING THE CONNECTION BETWEEN GESTURES AND LEARNING

Hand gestures are not just hand waving. We have now shown you that gestures enhance learning in children who view and make them. Furthermore, children’s gestures reveal when they are on the cusp of understanding a new concept. An important unanswered question that scientists are still trying to figure out is *how*? How do gestures help learning?

Even though we do not yet have an answer to this, scientists have some ideas. One possibility is that gestures offer a visual way to communicate ideas that can complement what children hear or say with spoken language. Instead of just hearing something in speech, children get to *see* it as well. Another possibility is that gestures help children focus their attention on the most important points of what is being learned, at exactly the right time. For example, when children are learning about mathematical equivalence and are being told, “*this side is equal to this side*,” the teacher’s gestures can bring children’s attention to the relevant pieces as they talk. Gestures increase the chance that the children will know exactly which part of the equation the teacher is talking about. Finally, it is also possible that gestures help memory by engaging more parts of the brain. Gesturing engages the motor (movement) parts of the brain in addition to the parts of the brain already active for producing language. Engagement of multiple brain areas may lead to better, deeper learning.

These unanswered questions are exciting opportunities for scientists to better understand how we learn and remember. Gestures come along for free with our speech and can play a critical role in helping children learn. By continuing to study gestures, we will better understand how we can help children learn both in the classroom and out.

Have you noticed your teachers gesturing in class? If not, it is time to tell them to start!

## Abbreviations

CO-SPEECH GESTURES: Gestures that we produce spontaneously while talking.
ICONIC GESTURES: Hand movements that create pictures to describe objects or actions.
BEAT GESTURES: Repeated hand movements that follow the rhythm of speech.
DEICTIC GESTURES: Gestures that direct the listener’s attention, such as pointing.
META-ANALYSIS: A mathematical approach that combines the results of many studies investigating similar questions, to make more powerful conclusions.
EQUIVALENCE: Having the same value or being equal. For example, in a math equation, the value of the left side of the equal sign is equivalent to the value of the right side.
